# Polymorphism of the *Prolactin* (*PRL*) Gene and Its Effect on Milk Production Traits in Romanian Cattle Breeds

**DOI:** 10.3390/vetsci10040275

**Published:** 2023-04-04

**Authors:** Daniela Elena Ilie, Alexandru Eugeniu Mizeranschi, Ciprian Valentin Mihali, Radu Ionel Neamț, Ludovic Toma Cziszter, Mihai Carabaș, Andrei Cristian Grădinaru

**Affiliations:** 1 The Research Department, Research and Development Station for Bovine Arad, 310059 Arad, Romania; 2 Department of Life Sciences, Faculty of Medicine, “Vasile Goldiș” Western University of Arad, 310025 Arad, Romania; 3 Faculty of Bioengineering of Animal Resources, University of Life Sciences ‘King Mihai I’ from Timișoara, 300645 Timișoara, Romania; 4 Department of Computer Science, Politehnica University of Bucharest, 060042 București, Romania; 5 Department of Preclinics, Faculty of Veterinary Medicine, “Ion Ionescu de la Brad” University of Life Sciences of Iași, 700489 Iași, Romania

**Keywords:** marker assisted selection, prolactin, Romanian Brown, Romanian Spotted

## Abstract

**Simple Summary:**

The analysis of the polymorphism of different loci with the potential to be involved in the pathway of obtaining different animal productions, such as he prolactin (PRL) gene for milk production, is of high interest and has effectiveness in the selection of animals and the improvement of livestock based on genetic markers.

**Abstract:**

In the present study, we investigated one polymorphism of the *PRL* gene (rs211032652 SNP) and assessed its influence on milk production and chemical composition in two Romanian cattle breeds. A total of 119 cattle from two breeds reared in Western Romania (64 Romanian Spotted and 55 Romanian Brown) were included in the research herd. A PCR-RFLP genotyping assay was used for the identification of the rs211032652 SNP variants. Shapiro’s test and Levene’s test were used to verify ANOVA assumptions and ANOVA and Tukey’s test were employed to test the associations between *PRL* genotypes and five milk traits. Among the studied breeds, our results showed that *PRL* genotypes were significantly associated (*p* < 0.05) with fat and protein percentage in the milk of Romanian Brown cattle. The *AA* genotype was associated with a higher fat percentage in milk (4.76 ± 0.28) compared to the *GG* genotype (4.04 ± 0.22, *p* = 0.048), as well as a higher protein percentage (3.96 ± 0.32% vs. 3.43 ± 0.15%, *p* = 0.027) in Romanian Brown cattle. Moreover, the *PRL* locus favored a significantly higher fat (*p* = 0.021) and protein (*p* = 0.028) percentage in the milk of Romanian Brown cattle compared to the Romanian Spotted breed, with a difference of 0.263% and 0.170%, respectively.

## 1. Introduction

Milk production is an important quantitative trait in cattle breeding, much improved in modern times by the selections made in livestock. As with any trait, it is a result of interactions of several genetic and environmental factors (e.g., food, additives, treatments, sheltering, etc.) [1].

The polymorphism of various candidate genes has been investigated in the past years, especially in terms of the association of their allelic variants with certain milk production traits (milk yield, protein and fat yields, protein and fat percentages) [2,3,4]. A previous study of the bovine prolactin (*PRL*) gene was conducted [5] revealing its position on the 23rd chromosome, with a spanning of 9.4 kb, containing five exons and four introns. It is not included in any linkage group quantitative trait loci (QTL) and it is not assumed to be a locus with a strong or weak effect on milk production traits. Instead, the gene of the prolactin receptor *(PRLR)* was demonstrated as having a weak effect in this regard [5].

*PRL* plays an important role in the initiation and maintenance of lactation. Various milk components, including proteins, lactose, lipids, and other important constituents, are synthesized as a result of its action at the level of mammary alveoli [5,6,7,8,9,10,11]. Milk synthesis is also supported by *PRL* as a result of dry matter intake and the increasing of feed intake [12]. Moreover, *PRL* has an indirect intervention at the level of milk protein gene expression [13,14].

Various polymorphisms were found in the bovine *PRL* gene [10] and 45 SNPs lying within exons have been reported in the Ensembl Variation database [15]. Although some of these are silent point mutations without any change in the encoded amino acid sequence [16,17], studies of bovine *PRL* genotype associations with milk traits were performed showing strong marker effects due to linkage with other polymorphisms [18]. Some of the investigated polymorphisms, such as the *Rsa*I site in exon 3, were also found in buffalo (*Bubalus bubalis*) and zebu (*Bos indicus*), thus being considered ancient polymorphisms, although independent origins of the mutations in the multiple mentioned species cannot be excluded [11,19]. For example, a silent *A*→*G* mutation in the codon responsible for amino acid 103 (A103G, which results in two alleles *A* and *B*), leads to a polymorphism which is frequently investigated in studies of genetic associations with quantitative milk traits. The *A* allele and the *AA* genotype at this locus were the most frequent in various cattle breeds, while the *BB* genotype was reported with the least frequency in the studied populations. An association of the *AA* genotype with milk yield was also reviewed, but with other contradictory results in this regard, considering the investigated breed or the number of animals [5].

In this work, we aimed to study the rs211032652 polymorphism in the bovine *PRL* gene [20] and to assess its influence on milk production and chemical composition in two populations of Romanian Spotted (Romanian Simmental) and Romanian Brown (Romania Brown of Maramureș) cattle breeds. This variant was previously found to be associated with milk production traits in multiple species, including *Bos taurus* [20]. This work is important, considering the general lack of research carried out on this polymorphism and the significance of the investigated cattle breeds in the context of the total livestock population of cattle in Romania. Both investigated breeds are among the predominant cattle breeds in Romania. The Romanian Spotted breed represents nearly 36% of the country’s overall population of cattle and is a dual-purpose breed developed as a result of non-systematical crossings between native Romanian Grey cows with imported bulls of the Simmental breed. Similarly, the Romanian Brown is another dual-purpose breed, for which the milk production traits are currently a top priority [4].

## 2. Materials and Methods

### 2.1. Animals and DNA Extraction

**Figure 1:**
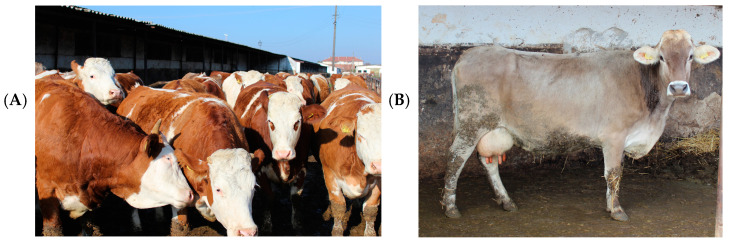
(**A**) Romanian Spotted breed; (**B**) Romanian Brown breed.

This research was conducted on 119 dairy cattle, of which 64 were Romanian Spotted (RS) and 55 belonged to the Romanian Brown (RB) breed (Figure 1). All animals belonged to the Research and Development Station for Bovine, located in Arad, Romania. Blood samples were collected from the tail vein in tubes containing K3EDTA as anticoagulant. After collection, the samples were stored at 4 °C, up until the DNA isolation. Genomic DNA was extracted using the manual kit Wizard Genomic DNA Purification (Promega, Madison, WI, USA). After extraction, the quality and quantity of DNA were assessed using a spectrophotometer (NanoDrop-2000, Thermo Fisher Scientific, Waltham, MA, USA) and by agarose gel electrophoresis with a concentration of 0.8%. Finally, the DNA samples were diluted to 50–100 ng and stored in the freezer at −20 °C prior to genotyping.

### 2.2. Phenotypic Records

The dairy cattle investigated in the study were included in the Romanian Official Performance and Recording Scheme. All dairy cattle participating in the study were kept in loose housing barns (in groups of 40 to 50 animals) with deep straw bedding and free access to outside paddocks, were fed twice per day, and had unlimited access to water. Daily individual diets consisted of 35 kg maize silage and 6 kg alfalfa hay supplemented with 6 kg concentrates. The cattle were milked two times per day in a “herringbone” milking parlor and milk records were taken every 28 days. Only cows with available data for the second lactation were included in the statistical analysis. Milk production data were available for a total number of 51 animals (29 Romanian Brown and 22 Romanian Spotted). The lactation records of cows consisted of normalized values for 305 days standard lactation length and mature equivalent, so that yields of different cows could be compared. The normalized data were obtained from the milk recording database of national farmer associations specific to the two breeds involved in the study. The investigated phenotypic traits were as follows: milk yield (kilograms), fat and protein yield expressed in kilograms, and the milk’s fat and protein percentages. Milk records were conducted by the Romanian Official Dairy Control service. The milk’s composition was determined by using CombiFoss integrating MilkoScan and Fossomatic instruments in the laboratory of the Milk Quality Control Foundation (Cluj-Napoca, Romania). The laboratory is accredited to the ISO standard (SR EN ISO/IEC 17025: 2018) for official milk performance analysis in Romania and the analytical instruments used by the laboratory are periodically calibrated.

### 2.3. Genotyping

The investigated polymorphism (ID SNP: rs211032652; g.35333764C>T; c.396G>A) in exon 4 of the *PRL* gene involves a transition of a *G* into an *A* nucleotide, which generates a restriction site for the *Rsa*I enzyme. According to the last updated information, this is a silent mutation which involves the amino acid 132, which is Valine for both variants (genome assembly ARS-UCD1.2). The polymorphism of rs211032652 in exon 4 of the *PRL* gene was analyzed using the PCR-RFLP technique. PCR amplification was carried out in a total volume of 25 μL reaction containing Green PCR Master Mix (Rovalab GmbH, Teltow, Germany), 20 pM of each forward and reverse primers [20] and approximately 50-100 ng of genomic DNA using a C1000 PCR Thermal Cycler (Bio-Rad Laboratories, Inc., Hercules, CA, USA). The PCR setup consisted of the following steps: an initial denaturation at 95 °C for 5 min, 35 cycles of denaturation at 95 °C for 30 s, annealing at 57 °C for 30s, and extension at 72 °C for 30 s, followed by one final extension of 72 °C for 5 min. Genotype identification was performed using the RFLP technique, where the PCR amplicons were subjected to restriction for two hours at 37 °C with the *Rsa*I restriction enzyme (Roche Diagnostics GmbH, Mannheim, Germany). Following digestion, the PCR-RFLP fragments were visualized in 4% agarose gel stained with Midori Green Advance dye (Nippon Genetics, Tokyo, Japan). The restriction pattern was observed under UV light using the UVP GelStudio PLUS system (Analytik Jena GmbH, Jena, Germany). The primer sequences used for amplification of exon 4 of the *PRL* gene, the PCR annealing temperatures of the forward and reverse primers, the expected size of the PCR amplicons, the restriction endonuclease used, and the corresponding genotypes are listed in Table 1.

### 2.4. Data Analysis

All data for milk quality traits (fat and protein yield expressed in kg, and the milk’s fat and protein percentages) and the single production trait (milk yield expressed in kg) were statistically analyzed and expressed as mean ± standard deviation (SD). The R package psych v. 1.9.12.31 [21] was used to compute several descriptive statistics as follows. A custom script written for the R programming environment v. 4.1.2 [22] was used to compute genotypic and allelic frequencies. The ggpairs function from the R package GGally v.2.0.0 [23] and the pairs.panels function from the R package psych were used to create scatterplots and compute correlations for the whole dataset and, individually, for the two investigated breeds. Deviations from Hardy–Weinberg equilibrium (HWE) were checked for both breeds included in this study using the chi-square test (X^2^). ANOVA and Tukey’s test were employed to carry out a statistical analysis using the corresponding base R methods to describe the effect of the three *PRL* genotypes on the milk production level, fat, and protein yield expressed in kilograms, as well as milk fat and protein percentages. Assumptions for using ANOVA were tested via the R functions shapiro.test from the base R package stats and leveneTest from the R package car v. 3.0-12 [24]; all the conditions were satisfied. Associations with *p* values less than 0.05 were considered significant.

### 2.5. Ethics Statement

All the involved research activities were carried out according to the Directive 2010/63/UE of the European Union’s Directive for animal experimentation. The planning of the experiments, collection of biological samples, experimental protocols, and procedures were approved by the Institutional Ethics Committee of the Research and Development Station for Bovine through the decision no. 75.

## 3. Results

### 3.1. Descriptive Statistics

A total number of 51 cows with lactation records for 305 days’ standard lactation length and mature equivalent were used in this study. The descriptive statistics of the observed phenotypes for each breed and milk production and quality traits are presented in Table 2.

**Figure 2:**
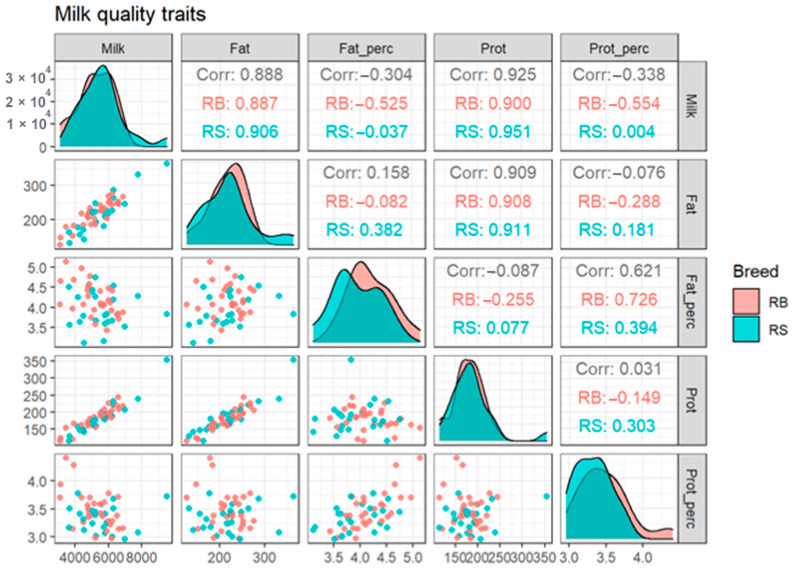
Scatterplots and correlations for the two breeds taken together and, individually, for each breed-specific population. The three values from each cell of the upper triangular portion of the figure represent the correlation between data points of pairs of phenotypes among the total population (RS + RB), and separately for the RB and RS breed-specific population, respectively. Corr, correlation; RB, Romanian Brown; RS, Romanian Spotted.

According to our results, the averages of milk, fat, and protein yield among breeds were 5580.43, 219.09 and 185.74 in RS and 5214.07, 216.76 and 179.59 in RB cattle, respectively. The overall mean and SD of fat and protein percentage were 3.93 ± 0.44 and 3.33 ± 0.24 for RS and 4.20 ± 0.42 and 3.38 ± 0.34 in RB cattle. For the observed phenotypes for each breed, we created scatterplots and computed correlations for the whole dataset and, individually, for the two investigated breeds (Figure 2). It can be observed that for fat and protein percentage there is a small, but visible difference in the corresponding histograms of the two breeds, which are represented in the diagonal of Figure 2.

### 3.2. Genotyping

**Figure 3:**
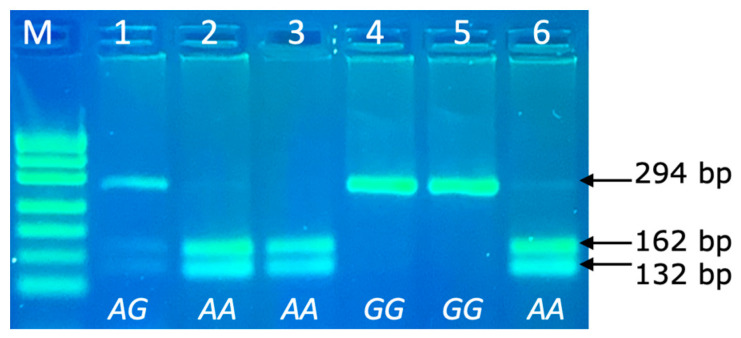
PCR-RFLP of *PRL* 294bp/*Rsa*I genotypes (rs211032652 SNP) in agarose gel electrophoresis. Line M: pUC19/Msp I Ladder (Carl Roth); Line 1: genotype *AG* (294, 162 and 132 bp); Lines 2, 3, 6: genotype *AA* (162 and 132 bp); Lines 4–5: genotype *GG* (294 bp).

The *PRL* genotypes were determined via the PCR-RFLP technique, with the following separation of allele specific fragments in 4% agarose gel. The PCR product size of *PRL* was 294 bp and the following DNA restriction fragments were obtained after RFLP: 162 and 132 bp for the *AA* genotype; 294, 162 and 132 bp for the *AG* genotype and 294 bp for the *GG* genotype (Figure 3).

### 3.3. Allele Frequencies

Genotype and allele frequencies of the *PRL* locus, expressed for each investigated breed and at the level of the entire population, are shown in Table 3. The chi-square test showed that both populations were in Hardy–Weinberg equilibrium (*p* > 0.05) for the *PRL* gene. Within the investigated breeds, a higher frequency of the *G* allele (0.9609) compared to the *A* allele (0.0390) was recorded in the RS breed. Slightly more similar values were obtained for the two allele frequencies in the RB breed, 0.4636 for *A* when compared to 0.5363 for the *G* allele. The *GG* homozygous genotype prevailed in the RS breed (0.9218) compared to *AG* (0.0781), while the *AA* homozygous genotype was entirely absent. For the RB breed, a higher frequency was found for the *AG* heterozygous genotype (0.4909) compared to homozygous *AA* (0.2181) and *GG* (0.2909), respectively.

### 3.4. Association Study

The effects of *PRL* genotypes on milk production and chemical composition in the RS and RB cattle breeds and the entire two-breed population (RS + RB) are listed in Table 4. Regarding the milk yield, no significant differences were recorded related to the genetic structures identified in the *PRL* locus. Thus, the highest milk production (5475.11 ± 1107.63 kg) was associated with the *GG* genotype for the RS breed. For the RB breed, the heterozygous *AG* genotype proved to be the genetic structure with the highest productive potential related to milk yield (5458.86 + 926.54 kg). No statistically significant differences were found in the assessment of the productive differences related to the three genetic structures, for the two studied breeds. The RS breed highlighted a low productive threshold associated with the *AG* heterozygous genotype, while the *AA* homozygous genotype showed a slightly higher level of milk production. Compared to the *GG* homozygous genotype which is considered the favorable genetic structure in order to obtain the highest milk production, no significant differences (*p* > 0.05) were calculated for the *AG* and *AA* genotypes. A similar situation was recorded for the RB breed in terms of the statistical association of productive thresholds with genetic structures identified in the *PRL* locus. The *AA* homozygous genotype was associated with reduced milk production (4380.25 ± 1327.29 kg), while a slightly increased production was recorded in the case of the *GG* homozygous genotype (5111.12 ± 1070.58 kg). Although productive differences compared to the favorable *AG* genotype were observed mathematically, they did not prove to be statistically significant.

The analysis of the total fat production (kg) highlighted a consistent trend of this parameter related to the cows’ genetic structure, a situation found in both breeds included in the study. The RS cows showed a high fat yield (247 kg) for the *AA* homozygous genotype, an intermediate yield for the *GG* homozygous genotype (202.00 ± 39.89 kg), and a lower production level associated with the *AG* heterozygous genotype, respectively. Despite an increased production by 22.27% and 14%, respectively, compared to the thresholds associated with the *AG* and *GG* genotypes, the productive capacity of the *AA* genotype does not generate significant differences (*p* > 0.05). For the RB breed, the maximum fat yield was associated with the *AG* heterozygous genotype (223.76 ± 30.11 kg). The *AA* and *GG* homozygous genetic structures recorded comparable, almost identical productive levels (206.75 ± 54.44 vs. 206.88 ± 45.19, *p* > 0.05). The productive differences calculated between the three genotypes proved to be verified from a mathematical point of view, without generating statistically significant differences (*p* > 0.05).

Given that milk yield and fat content (%) had a negative correlation, the dynamics of these traits were examined with respect to breed and the genetic structures of the *PRL* locus. The analysis performed within the RS herd did not highlight statistically significant differences between the genotypes. The correlation between the milk yield and fat content was verified from a statistical point of view. As in the case of milk yield, the genotype did not significantly influence the fat content (*p* > 0.05). The highest value for fat content was recorded for the *AA* homozygous genotype (4.75%), while the minimum threshold was associated with the GG genotype (3.88 ± 0.54%), which is favorable to milk production. For the RB breed, different levels of milk fat content related to the cows’ genetic structure were recorded. The genotype associated with lower milk yield (*AA*) allows a higher fat content (4.76 ± 0.28%). Compared to the heterozygous *AG* and homozygous GG genotypes, the *AA* genotype induces statistically significant differences (*p* < 0.05). Comparing the estimated productive gain relative to the values associated with *AG* and *GG* genotypes, it reached thresholds of 14.97% and 17.8%, respectively.

The protein yield (kg) did not prove to be influenced by the cows’ genetic structure, the situation being similar in both studied breeds. Although the *AA* homozygous genotype proved to be the favorable genotype for protein production (197 kg) in the RS breed, the calculated differences compared to the *AG* (174.33 ± 27.10 kg) and *GG* (178.67 ± 34.15 kg) genotypes were statistically insignificant (*p* > 0.05). This situation was also found for the RB breed. The *AG* heterozygous genotype was favorable for protein production (183.64 ± 28.54 kg). The homozygous structures recorded reduced and comparable protein yields (171.25 ± 43.85 kg vs. 174.75 ± 33.33 kg for *AA* and *GG* genotypes, respectively), both among themselves and relative to the favorable *AG* genotype. The productive gain associated with the *AG* genotype generated only mathematical differences, no statistical significance being observed in this respect (*p* > 0.05).

Within the RS herd, the highest protein content was associated with the *AG* heterozygous genotype (3.46 ± 0.10%), which proved to be totally unfavorable for milk production, as we previously observed. The lower protein threshold was associated with the homozygous *GG* genotype (3.28 ± 0.24%). An intermediate level of protein content was calculated for the *AA* genotype (3.44%). The overall analysis of these associations reveals the lack of a significant influence of the cows’ genotype on the milk protein content (*p* > 0.05). Within the RB breed, the correlation with the milk yield was also verified. Furthermore, a significant influence of the genotype on the milk protein content was recorded (*p* < 0.05). The higher protein content threshold was associated with the *AA* genotype (3.96 ± 0.32%), which generates significant differences (*p* < 0.05) compared to the related *AG* (3.39 ± 0.33%) and *GG* (3.43 ± 0.15%) genotypes productive levels.

During our evaluation of the effect of *PRL* genotypes on the milk yield, fat, and protein yield, as well as fat and protein percentages of milk in the entire two-breed population (RS + RB), we identified a statistically significant difference between genotypes in the case of the fat and protein percentages in milk (*p* < 0.05). Cows carrying the *AA* genotype had a higher fat percent (4.76 ± 0.24%) than those with *AG* (4.12 ± 0.40%) or *GG* (3.93 ± 0.40%) genotypes. The same results were also obtained in relation to protein percentage (*p* < 0.05), where animals with the *AA* genotype had a significantly higher milk protein content (3.85 ± 0.36%) than the *AG* (3.40 ± 0.31%) or *GG* (3.32 ± 0.22%) cows. Furthermore, no significant differences (*p* > 0.05) were observed between the *PRL* genotypes and milk yield or fat and protein yield expressed as kilograms (*p* > 0.05).

In conclusion, the RB cattle showed a significantly higher fat (*p* = 0.021) and protein (*p* = 0.028) percentage in milk compared to the RS breed, with a difference of 0.263% and 0.170%, respectively. Considering that the environmental effects (housing and feeding conditions) were identical for animals from both breeds, we can conclude that the observed phenotypic differences are due to the genetic make-up of the two breeds.

## 4. Discussion

Prolactin is a chemical mediator consisting, in the majority of mammalian species, of 197–199 amino acids. Its synthesis and secretion at both the central and local systems have a well-established impact on the milk production pathway. We previously reviewed the mechanisms for physiological action of the PRL hormone and described the main site of its synthesis and secretion as the prolactin cells of the anterior pituitary (~60% of polysomal mRNA found at this level), although the mRNA product of this gene was also detected in other tissues, such as brain, mammary gland, those of reproduction (placenta, amnion, uterus), and blood cells, such as lymphocytes. The PRL hormone plays an important role in mammogenesis, lactogenesis, and galactopoiesis, and is considered to be related to milk synthesis and secretion. Various polymorphisms on its coding gene were investigated in cattle over the time, in some cases with contradictory results depending on the investigated breed or number of animals [5].

The investigated polymorphism in this work has been considered in various reports. Boleckova et al. [18] investigated the same polymorphism in the Czech Fleckvieh cattle breed and reported the same predominance of the *G* allele compared to *A* allele as in our study, and the same predominance of the *GG* genotype compared to *AG* and *AA*, the last one having the lowest frequency. We recorded the same tendency of allele frequencies in RB cattle, but not at such amplitude, whereas in the matter of genotype frequencies, the *AG* genotype was predominant, while homozygous genotypes had almost the same frequencies. In the aforementioned work, the *G* allele of *PRL* was positively associated with milk yield, with a subsequent positive influence on protein and fat yields, but a negative influence on protein and fat percentages, probably as a consequence of the negative correlation between fat percentages and milk yield in cattle. Conversely, Mehmannavaz et al. [10] highlighted the *G* allele as unfavorable for milk and protein yields.

Our obtained results finding the *GG* genotype at the *PRL* locus more prevalent than the *AG* and *AA* genotypes in RS cattle, and the higher frequency of the *G* allele compared to the *A* allele in both investigated breeds were also confirmed in Holstein Friesian cattle, crossbred Angus-based cows, and Iranian Holstein [10,16,25], although none of those studies found a lack of the *AA* genotype in the investigated populations and such a large range of variation in terms of genotype frequencies as we found in the RS cattle population.

The results obtained in our work suggest that the polymorphism at the *PRL* locus has an effect on fat and protein percentages in the milk of analyzed RB cows, and not in RS cows. Statistical analyses showed that the *AA* genotype for *PRL* determines a significantly higher fat and protein percentage in milk. In our study, no significant differences (*p* > 0.05) were observed between the *PRL* genotypes and milk yield or fat and protein yield expressed as kilograms (*p* > 0.05). In previous studies, the *PRL* polymorphism was associated with higher milk yield [18,26]. However, these results can also be assigned to the number of investigated animals (n = 51), which was relatively small, and may reduce the statistical power of the association analysis. The analysis performed with respect to the milk protein content (%) highlights two aspects. On one hand, the strong and negative correlation between milk yield and its protein content is evident, a situation found in both studied breeds. On the other hand, the analysis performed highlights the reduced ability of the genotype to influence the protein content.

Investigating the same polymorphism in exon 4 of the *PRL* gene, Lü et al. [9] failed to demonstrate any association with milk performance traits in Chinese Holstein cattle, although other reports reviewed by the authors showed an association of the *AG* genotype in black and white cows with the highest milk yield, and of the *GG* genotype with the highest fat content. These results matched those found by us only in the case of the *AG* genotype associated with the highest milk yield in the RB breed. Moreover, the results obtained by Thuy et al. [16] highlighted no significant differences between the cows of different genotypes, where cows with the *PP* genotype (i.e., the *GG* genotype in our study) produced more milk compared to those with *PC* (*AG*) and *CC* (*AA*) genotypes. In a cited study by Meyer et al. [25], the homozygous *GG* cows were reported with more fat content, and the heterozygous (*AG*) cows had increased milk yield, results which correspond only with those obtained by us in the case of the superiority of the *AG* genotype for milk production in RB cattle.

## 5. Conclusions

In this study, we investigated a polymorphism within exon 4 of the bovine *PRL* gene (rs211032652 SNP, c.396G>A) and assessed the genotype influence on milk production and chemical composition in the RS and RB cows. The results obtained revealed that the most frequent genotypes in the RS and RB populations were *GG* (0.9218) and *AG* (0.4909), respectively. These findings agree with several previous researches on *PRL* polymorphism with respect to the allele and genotype frequencies. According to the statistical analysis, *AA* genotypes may have an influence on milk fat and protein percent (*p* < 0.05) among RB cattle. This work contributes to the literature with possible application in Romanian cattle breeding, where such types of studies need to be further developed. In conclusion, these results revealed the suitability of the rs211032652 SNP in the *PRL* gene for potential use in dairy cattle breeding as genetic marker for the milk quality management of Romanian Spotted and Romanian Brown cattle.

## Figures and Tables

**Table 1:** The forward and reverse primers used for the amplification of *PRL*, PCR amplicon product size, restriction endonuclease, and genotypes according to the obtained digestion fragments.

Gene	Primers (5′-3′)	Annealing Temp. (°C)	Amplicon Size (bp)	Restriction Endonuclease	Digestion Product Size (bp)
*PRL*	F: 5′- CCA AAT CCA CTG AAT TAT GCT T -3′;	57	294	*Rsa*I	(*AA*) 162,132
	R: 5′- ACA GAA ATC ACC TCT CTC ATT CA -3′				(*AG*) 294,162,132
					(*GG*) 294

**Table 2:** Descriptive statistics for milk production and quality traits in Romanian dairy cattle.

Breed	Trait	N	Mean	SD	Min	Max
RS	Milk (kg)	22	5580.43	1323.29	3623.00	9515.00
	Fat (kg)	22	219.09	56.17	131.00	364.00
	Fat (%)	22	3.93	0.44	3.11	4.75
	Protein (kg)	22	185.74	48.12	115.00	354.00
	Protein (%)	22	3.33	0.24	2.96	3.78
RB	Milk (kg)	29	5214.07	1050.97	3099.00	6883.00
	Fat (kg)	29	216.76	37.66	126.00	275.00
	Fat (%)	29	4.20	0.42	3.43	5.14
	Protein (kg)	29	179.59	31.25	115.00	244.00
	Protein (%)	29	3.48	0.34	2.97	4.41

RS, Romanian Spotted breed; RB, Romanian Brown breed; N, number of animals; SD, standard deviation; Min, minimum value; Max, maximum value.

**Table 3:** Allele and genotype frequency distributions for the *PRL* locus in the Romanian Spotted (RS) and Romanian Brown (RB) cattle breeds and the entire two-breed population (RS + RB).

Breed	n	Allele Frequency		Genotype Frequency (n)		
		*A*	*G*	*AA*	*AG*	*GG*
RS	64	0.0390	0.9610	0.00 (0)	0.0781 (5)	0.9219 (59)
RB	55	0.4636	0.5364	0.2181 (12)	0.4910 (27)	0.2909 (16)
Total	119	0.2353	0.7647	0.1008 (12)	0.2689 (32)	0.6303 (75)

n, number of animals.

**Table 4:** Mean ± standard deviation (SD) for milk production and chemical composition traits based on the genotypes of the *PRL* locus in Romanian Spotted (RS) and Romanian Brown (RB) cattle breeds.

Breed	Genotype (n)	Milk (kg)	Fat (kg)	Fat (%)	Protein (kg)	Protein (%)
RS	*AA* (1)	5200.00 ^a^	247.00 ^a^	4.75 ^a^	197.00 ^a^	3.44 ^a^
	*AG* (3)	5027.67 ± 680.48 ^a^	202.00 ± 39.89 ^a^	3.99 ± 0.32 ^a^	174.33 ± 27.10 ^a^	3.46 ± 0.10 ^a^
	*GG* (18)	5475.11 ± 1107.63 ^a^	212.33 ± 50.14 ^a^	3.88 ± 0.54 ^a^	178.67 ± 34.15 ^a^	3.28 ± 0.24 ^a^
RB	*AA* (4)	4380.25 ± 1327.29 ^a^	206.75 ± 54.44 ^a^	4.76 ± 0.28 ^b^	171.25 ± 43.85 ^a^	3.96 ± 0.32 ^b^
	*AG* (17)	5458.86 ± 926.54 ^a^	223.76 ± 30.11 ^a^	4.14 ± 0.41 ^a^	183.64 ± 28.54^a^	3.39 ± 0.33 ^a^
	*GG* (8)	5111.12 ± 1070.58 ^a^	206.88 ± 45.19 ^a^	4.04 ± 0.22 ^a^	174.75 ± 33.33 ^a^	3.43 ± 0.15 ^a^
Total (RS + RB)	*AA* (5)	4544.20 ± 1206.51 ^a^	214.80 ± 50.46 ^a^	4.76 ± 0.24 ^b^	172.80 ± 38.13 ^a^	3.85 ± 0.36 ^b^
	*AG* (20)	5394.05 ± 892.53 ^a^	220.50 ± 31.53 ^a^	4.12 ± 0.40 ^a^	182.40 ± 27.85 ^a^	3.40 ± 0.31 ^a^
	*GG* (26)	5363.12 ± 1088.36 ^a^	210.65 ± 47.84 ^a^	3.93 ± 0.40 ^a^	177.46 ± 33.28 ^a^	3.32 ± 0.22 ^a^

n, number of animals; Column means with different superscripts differ significantly at *p* ≤ 0.05, within the same source of variation.

## Data Availability

The data presented in this study are available on request from the corresponding author. The data are not publicly available due to privacy reasons regarding the phenotypic data, which are owned by the Romanian Breeding Association “Bălțată Românească” Simmental type (ACVBR-SIM Harman-Brasov, Romania) and Innovative Agricultural Services (Reading, UK).
